# The association between albuminuria and thyroid antibodies in newly diagnosed type 2 diabetes mellitus patients with Hashimoto’s thyroiditis and euthyroidism

**DOI:** 10.1186/s12902-020-00650-0

**Published:** 2020-11-23

**Authors:** Wei Zhu, Xuejie Dong, Qingrong Pan, Yanjin Hu, Guang Wang

**Affiliations:** 1 Department of Endocrinology, Beijing Chao-yang Hospital, Capital Medical University, Beijing, 100020 People’s Republic of China; 2 Department of Endocrinology, Beijing Aerospace General Hospital, Beijing, 100076 People’s Republic of China

**Keywords:** Type 2 diabetes mellitus, Hashimoto’s thyroiditis, Microalbuminuria

## Abstract

**Background:**

Microalbuminuria is a prognostic marker of diabetes kidney disease. It is generally diagnosed as the ratio of urinary albumin to creatinine (UACR) of 30-300 mg/g. Hashimoto’s thyroiditis is a common disease in the endocrinology and the thyroid antibodies may associated with kidney disease. We investigated the UACR in the newly diagnosed T2DM with Hashimoto’s thyroiditis and tried to detect the relationship between the UACR and thyroid antibodies.

**Methods:**

One hundred twenty newly diagnosed T2DM patients with Hashimoto’s thyroiditis and euthyroidism and 50 sex and age-matched T2DM with non-Hashimoto’s and other thyroid disease were recruited. T2DM patients were divided into 2 groups by the titer of TPOAb: (1). TPOAb (+) group: T2DM with positive TPOAb (*n* = 105); (2). TPOAb (−) group: T2DM with negative TPOAb (*n* = 65).

**Results:**

T2DM with positive TPOAb group had higher UACR than T2DM with negative TPOAb group (21.55 ± 7.28 vs 15.13 ± 5.69 mg/g, *P < 0.01*). UACR were positively related to BMI (*r* = 0.255, *P < 0.05*), FPG (*r* = 0.285, *P < 0.05*), HbA1c (*r* = 0.260, *P < 0.05*) and TPOAb (*r* = 0.349, *P < 0.05*). HbA1c (β = 0.793, *P < 0.05*), BMI (β = 0.342, *P < 0.05*) and lnTPOAb (β = 1.207, *P < 0.05*) were independently associated with UACR.

**Conclusions:**

In the newly diagnosed T2DM patients, Hashimoto’s thyroiditis with TPOAb positive had higher UACR levels. TPOAb titer, BMI and HbA1c were independent associated with UACR in these patients.

## Background

Type 2 diabetes mellitus (T2DM) is an important public health problem in the world and 10.9% of the adult population in China was affected in 2013 [1]. Microalbuminuria is generally diagnosed as the ratio of urinary albumin to creatinine (UACR) of 30-300 mg/g or urine albumin excretion of 30-300 mg/24 h [2]. Microalbuminuria is a prognostic marker of diabetes kidney disease [3]. In addition, microalbuminuria increased the risk of cardiovascular morbidity and mortality, stroke, and heart failure. It begins even when the microalbuminuria is in normal range or high-normal range in both diabetes and euglycemic individuals [4–6]. For the diabetes patients, American diabetes association suggested that the urinary albumin should be assessed at least once per year [7].

Hashimoto’s thyroiditis is another common disease in the endocrinology. The morbidity of Hashimoto’s thyroiditis is 0.2% in men and 2% in women [8]. It is an importance cause of hypothyroidism. The autoimmune mechanism of Hashimoto’s thyroiditis is associated to the renal disease. Some evidences point that about 10–30% Hashimoto’s thyroiditis patients had microproteinuria or nephrotic syndrome [9]. The possible mechanism is that thyroid antigens including thyroglobulin (TG) and thyroperoxidase (TPO) are released in the situation of Hashimoto’s thyroiditis. Both of TPO and TG can combine with their antibodies (TGAb and TPOAb) and form circulating immune complex. It can deposit in the glomerulus and as nephritis antigen to form in situ immune complex [10]. But whether Hashimoto’s thyroiditis aggravated the microalbuminuria in T2DM patients, the research is few. In this study, we tried to detect the association between the thyroid antibodies of Hashimoto’s thyroiditis and microalbuminuria in newly diagnosed T2DM with normal thyroid function patients.

## Methods

### Subjects

A total of 120 newly diagnosed T2DM patients with Hashimoto’s thyroiditis and euthyroidism and 50 newly diagnosed T2DM patients without Hashimoto’s and other thyroid disease were recruited in the outpatient endocrinology department of Beijing Chao-yang hospital from June 2015 to June 2018. Diagnostic criteria for Hashimoto’s thyroiditis were (1). The ultrasound tests showed thyroid parenchymal heterogeneity. (2). Elevated of thyroperoxidase antibody (TPOAb, >60 IU/ml) and/or thyroglobulin antibody (TGAb, >60 IU/ml). Diagnostic criteria of diabetes mellitus were according to the World Health Organization (WHO) criteria 2019. All patients should have the normal thyroid function. Their free T3 (FT3), free T4 (FT4), total T3 (TT3), total T4 (TT4) and thyroid-stimulating hormone (TSH) were in the normal range. All subjects were excluded if they had the history of other thyroid disease such as sub-acute thyroiditis, Graves’ disease and thyroid carcinoma, type 1 diabetes mellitus and renal disease. Other diseases affecting microalbuminuria such as hypertension, systemic lupus erythematosus, gout and urinary tract infection were excluded. T2DM patients without thyroid disease should had euthyroidism and normal TPOAb and TGAb level.

T2DM patients were divided into 2 groups by the titer of TPOAb: (1). TPOAb (+) group: T2DM with positive TPOAb (*n* = 105); (2). TPOAb (−) group: T2DM with negative TPOAb (*n* = 65).

All patients enrolled in the study gave informed consent. All procedures were conducted in accordance with Declaration of Helsinki. The ethics committee of Beijing Chao-yang Hospital approved the present research.

### Laboratory measurements

All 170 patients underwent the assessment of the physical examination including height, weight and blood pressure at the fasting state. Blood samples were collected at morning fasting state. Test items included fasting plasma glucose (FPG), HbA1c, total cholesterol (TC), low-density lipoprotein cholesterol (LDL-C), high-density lipoprotein cholesterol (HDL-C), triglycerides (TG), creatinine (Cr) and thyroid function (FT3, FT4, TT3, TT4. TSH), thyroid antibodies (TPOAb, TGAb). FT3, FT4, TT3, TT4, TSH, TPOAb and TGAb were measured by chemiluminescence. The normal ranges of thyroid parameters are listed below: FT3: 1.71–3.71 pg/ml; FT4: 0.7–1.48 ng/dl; TT3: 0.58–1.59 ng/ml; TT4:4.87–11.72μg/dl, TSH: 0.35–4.94 IU/ml, TPOAb: 0-60 IU/ml; TGAb: 0-60 IU/ml. Urinary albumin was tested using immunoturbidimetric assay. Urinary creatinine was enzymatically measured. Microalbuminuria was evaluated by the urinary albumin-to-creatine ratio (UACR) in a random spot urine collection (mg/g). Thyroid ultrasound was performed in each subject. Body mass index (BMI) was calculated as height (kg)/weight^2^(m^2^).

### Statistical analysis

Data were analyzed by SPSS 20.0 software (SPSS, Inc., Chicago, IL, USA). Continuous data as age, BMI, TC, LDL-C, HDL-C, Cr FT3, FT4, TT3, TT4, TSH, UACR, systolic pressure (SBP) and diastolic pressure (DBP) were expressed as Mean ± SD. Non-normally distributed variables as TG, TPOAb, TGAb were expressed as median (IQR). Continuous data were analyzed by Student’s t test. Non-normally distributed variables were analyzed by nonparametric test. Pearson or Spearman’s rank correlation was used to assess the association between UACR and other parameters. Multiple linear regressions were used to assess the associated factors of UACR. TPOAb was ln transformed before analysis. All analyses were two tailed and *P < 0.05* were considered statistically significant.

## Results

### Baseline characteristics of the newly diagnosed T2DM patients with and without positive TPOAb

The baseline characteristics of newly diagnosed T2DM patients were performed in Table 1. The age, sex, BMI, blood pressure, TC, HDL-C, HDL-C, TG, FPG, HbA1c, Cr, TT3, TT4, FT3, FT4, TSH were matched in the two groups (Table 1).

**Table 1:** Comparison of clinical parameters of T2DM with positive TPOAb and with negative TPOAb

	TPOAb (+) (***n*** = 105)	TPOAb(−) (***n*** = 65)	***P*** Value
**Age, years**	46.9 ± 10.8	48.2 ± 10.5	0.429
**Sex, male/female**	29/76	20/45	0.393
**BMI, kg/m**^**2**^	27.45 ± 4.80	27.04 ± 4.67	0.590
**SBP: mmHg**	115.9 ± 13.5	117.43 ± 13.23	0.466
**DBP: mmHg**	75.2 ± 8.9	77.2 ± 9.3	0.151
**TC, mmol/L**	5.30 ± 1.02	5.16 ± 1.23	0.451
**LDL-C, mmol/L**	3.07 ± 0.87	2.92 ± 0.80	0.258
**HDL-C, mmol/L**	1.23 ± 0.35	1.18 ± 0.31	0.310
**TG, mmol/L**	1.61 (1.06,2.82)	1.53 (1.09,2.57)	0.941
**Cr, umol/L**	69.80 ± 13.30	67.36 ± 12.96	0.661
**FPG, mmol/L**	8.88 ± 2.93	8.62 ± 1.92	0.533
**HbA1c: %**	8.85 ± 1.76	8.55 ± 1.60	0.250
**TT3: ng/ml**	0.96 ± 0.21	1.02 ± 0.23	0.069
**TT4: ug/dl**	6.33 ± 1.33	6.71 ± 1.60	0.095
**FT3: pg/ml**	2.52 ± 0.43	2.64 ± 0.48	0.090
**FT4: ng/dl**	1.10 ± 0.16	1.15 ± 0.23	0.133
**TSH: Uiu/ml**	2.06 ± 1.25	2.09 ± 0.84	0.869
**TPOAB: IU/ml**	246.19 (118.37,621.80)	22.74 (8.29, 33.43)	< 0.001
**TGAB: IU/ml**	71.55 (24.25, 188.74)	20.04 (8.97,47.84)	< 0.001

Abbreviations: *BMI* body mass index; *SBP* systolic pressure; *DBP* diastolic pressure; *TC* total cholesterol; *LDL-C* low-density lipoprotein cholesterol; *HDL-C* high-density lipoprotein cholesterol; *TG* triglycerides; *Cr* creatinine; *TT3* total T3; *TT4* total T4; *FT3* free T3; *FT4* free T4; *TSH* thyroid stimulating hormone; *TPOAb* thyroperoxidase antibody; *TGAb* thyroglobulin antibody

**Fig. 1:**
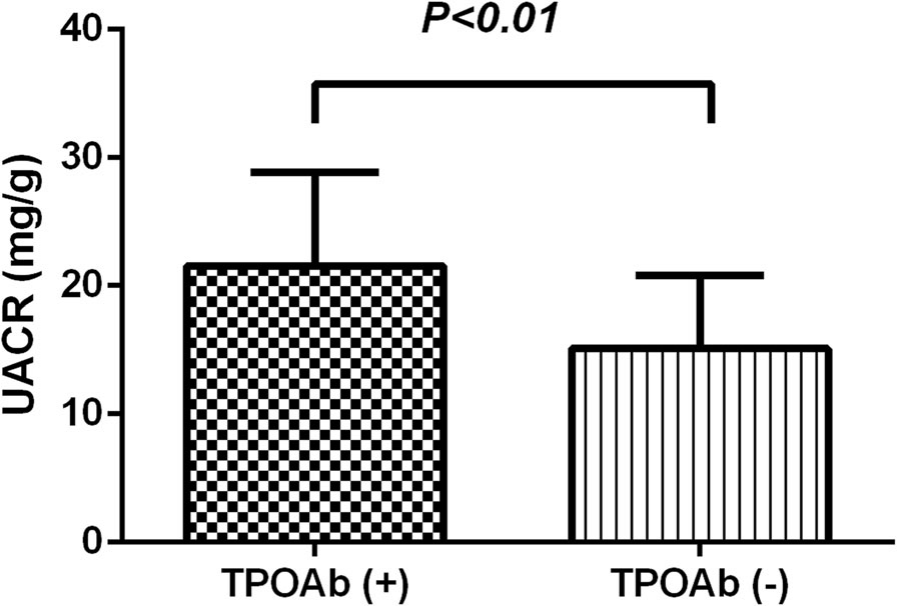
UACR values of newly diagnosed type 2 diabetes mellitus subjects with TPOAb positive and TPOAb negative group. UACR values were expressed as mean ± SD, and *P < 0.05* was considered statistically significant. TPOAb (+): T2DM with TPOAb positive. TPOAb (−): T2DM with TPOAb negative. Abbreviations: UACR: ratio of urinary albumin and creatinine; T2DM: type 2 diabetes mellitus; TPOAb: thyroid peroxidase antibody; TGAb: thyroglobulin antibody

The differences of UACR were summarized in Fig. 1. TPOAb positive group had higher UACR than TPOAb negative group (21.55 ± 7.28 vs 15.13 ± 5.69 mg/g, *P < 0.01,* Fig. 1).

### The correlations between the UACR and other parameters

**Fig. 2:**
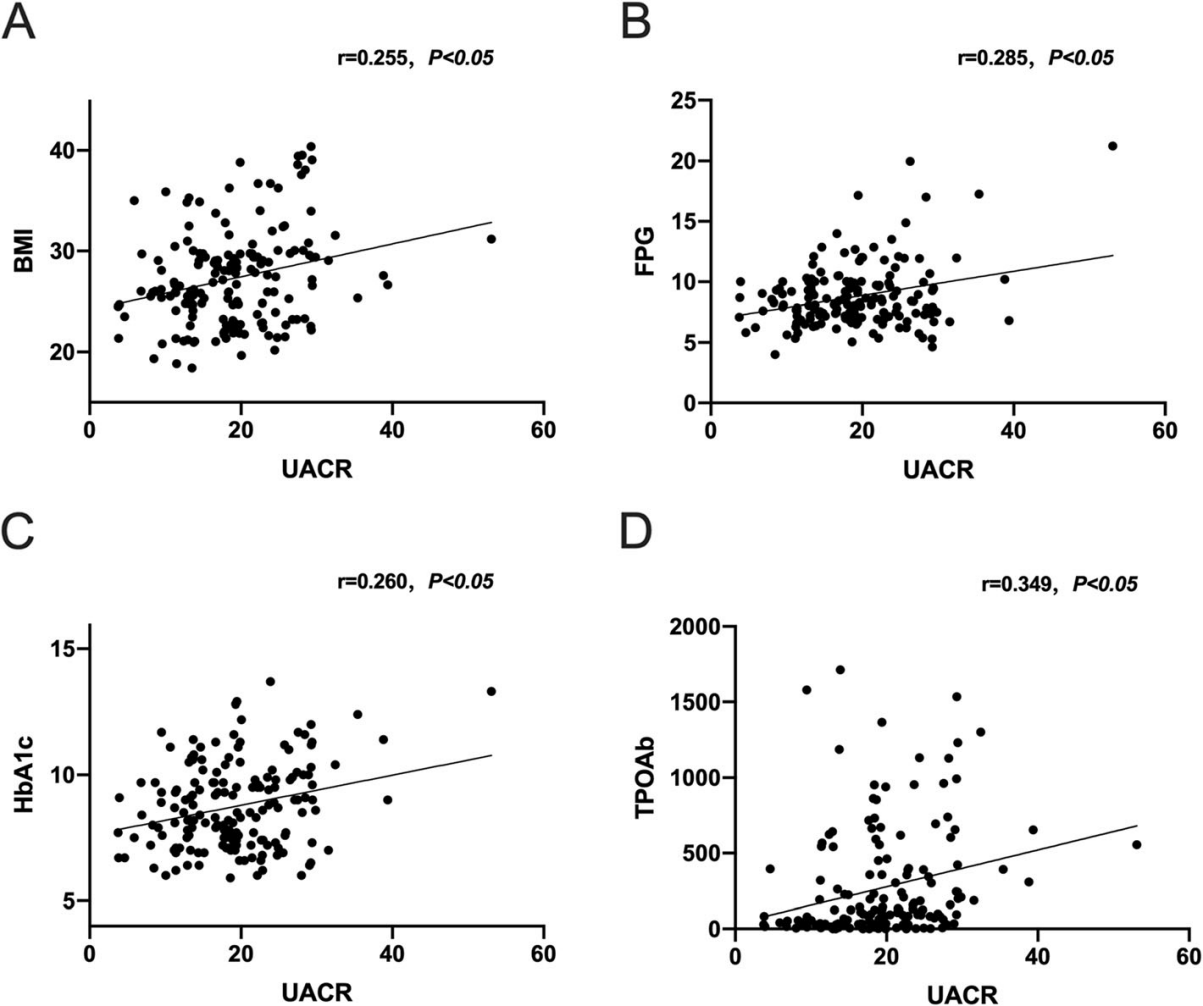
UACR were positively related to the BMI (*r* = 0.255, *P < 0.05,* **a**, FPG (*r* = 0.285, *P < 0.05,* **b**, HbA1c (*r* = 0.260, *P < 0.05,* **c** and TPOAb (*r* = 0.349, *P < 0.05,* **d**. Abbreviations: UACR: ratio of urinary albumin and creatinine; BMI: body mass index; FPG: fasting plasma glucose; TPOAb: thyroid peroxidase antibody; TGAb: thyroglobulin antibody

The correlation analyses were conducted to test the associations between UACR and other parameters (Fig. 2a-d). UACR were positively related to the BMI (*r* = 0.255, *P < 0.05,* Fig. 2a), FPG (*r* = 0.285, *P < 0.05,* Fig. 2b), HbA1c (*r* = 0.260, *P < 0.05,* Fig. 2c) and TPOAb (*r* = 0.349, *P < 0.05,* Fig. 2d). The relationships of UACR and other parameters as age, sex, SBP, DBP, TC, LDL-C, HDL-C, TG, TT3, TT4, FT3, FT4, TSH, TGAb were not significant (*P>0.05*).

### Multiple regressions of UACR and its related factors

Table 2 showed the multiple regressions of various independent variables to test the association with UACR. HbA1c, BMI and TPOAb were entered in the regression model. TPOAb was Ln transformed before analysis. Multiple regression analysis demonstrated that HbA1c (β = 0.793, *P < 0.05*), BMI (β = 0.342, *P < 0.05*) and ln TPOAb (β = 0.1.207, *P < 0.05*) were independently associated with UACR.

**Table 2:** Multiple stepwise regression analysis of the parameters associated with RHI

Parameters	Β	SE	P value
BMI, kg/m2	0.342	0.112	0.003
HbA1C, %	0.793	0.313	0.014
Ln-TPOAb, IU/ml	1.207	0.287	< 0.001

Abbreviations: *BMI* body mass index; *TPOAb* thyroperoxidase antibody

## Discussion

UACR is the ratio of urinary albumin and creatinine. It represents the microalbuminuria in the T2DM. UACR is not only the important marker of diabetes kidney disease with diabetes kidney disease which occurs in nearly 20–40% of diabetes patients, but also the predictor of some complications of T2DM such as cardiovascular disease and stroke and so on [5]. In the previous studies, the morbidity and mortality of cardiovascular was positively associated with UACR, although the UACR is in the normal range [5, 11]. These effects even happen in the non-diabetes [4]. In our research, the UACR of newly diagnosed T2DM patients was positively related to the fasting plasma glucose and HbA1c. In the research of diabetes, there is a clear relationship between the microalbuminuria and glycemic control. Intensive glycemic control has been shown in many studies to delay the onset and progression of albuminuria in both type 1 and type 2 diabetes mellitus [12, 13]. Our findings were consistent with those previous studies.

The main finding of our research is the relationship between the TPOAb and albuminuria in newly diagnosed T2DM patients. In our research, T2DM with TPOAb positive patients had higher albuminuria than TPOAb negative patients. In the correlated and regression analysis, TPOAb seem to be the main factor related to the albuminuria in T2DM patients. In the previous studies, thyroid function is closely related to the albuminuria [14]. Both hypothyroidism and subclinical hypothyroidism could aggravate albuminuria in the T2DM patients [15]. But in the Hashimoto’s thyroiditis with euthyroidism the research is few. In our study, we excluded the effects of thyroid hormone and thyroid simulate hormone on albuminuria. The difference of albuminuria may be due to the autoimmune mechanism. In 1970s, it was reported that patients with Hashimoto’s thyroiditis might be accompanied with proteinuria [16]. Some researchers found that TPO and TG in the subepithelial immune deposit and mediated immune complex glomerulonephritis. Both of TPO and TG can be trapped in subendothelial level and elevated glomerular permeability [17]. In addition, Guangda Xiang’s research demonstrated that Hashimoto’s thyroiditis patients had endothelial serious dysfunction even in the stage of euthyroidism. Endothelial dysfunction is associated with albuminuria [18].

In the histopathology research both TPOAb and TGAb can be trapped at subendothelial level. But in our research, we found TPOAb was the independent influence factor of UACR but not TGAb. In addition, the differences of TPOAb and TGAb are in the complement system and cytotoxicity. TPOAb could induce the complement system and cellular cytotoxicity in contrast to TGAb. The effects lead to the aggravation of inflammatory status [19].. It may result in the higher UACR level in the T2DM patients.

The present research also found that BMI is the independently associated with UACR. This is consistent with many previous studies. Obesity especially visceral obesity is the important risk factor of diabetes kidney disease [20, 21]. One possible hypothesis is obesity-induced glomerular hyperfiltration and increased urinary albumin excretion rate [22]. Moreover, adipocyte secrete inflammatory factors such as TNF-α and C-reactive protein. These factors are toxic to glomerular podocytes and mesangial cells [23, 24]. Other mechanisms include insulin resistance, excessive lipid deposition oxidative stress caused by obesity [25, 26].

Some limitations of the present study must be mentioned. First, the sample size of Hashimoto’s thyroiditis patients with T2DM was small. Second, the diagnosis of Hashimoto’s thyroiditis accorded to the TPOAb, TGAb and ultrasound test. Pathological biopsy might make the diagnosed of Hashimoto’s thyroiditis more accurate. Finally, the UACR is influenced by many factors such as glucose, blood pressure, exercise within 24 h, menstruation and so on. The reexamine in another day or 24 h may make the results more credible.

## Conclusions

In the newly diagnosed T2DM patients, Hashimoto’s thyroiditis with TPOAb positive had higher UACR levels. TPOAb titer, BMI and HbA1c independent associated with UACR in these patients.

## Data Availability

The datasets used and/or analyzed during the current study are available from the corresponding author on reasonable request.

## Abbreviations

T2DM: Type 2 diabetes mellitus
TPOAb: Thyroperoxidase antibody
TGAb: Thyroglobulin antibody
UACR: Ratio of urinary albumin and creatinine
BMI: Body mass index
WHO: World Health Organization
FT3: Free T3
FT4: Free T4
TT3: Total T3
TT4: Total T4
TSH: Thyroid-stimulating hormone
FPG: Fasting plasma glucose
TC: Total cholesterol
LDL-C: Low-density lipoprotein cholesterol
HDL-C: High-density lipoprotein cholesterol
TG: Triglycerides
